# Investigation of Carbon-Based Composites for Elastic Heaters and Effects of Hot Pressing in Thermal Transfer Process on Thermal and Electrical Properties

**DOI:** 10.3390/ma14247606

**Published:** 2021-12-10

**Authors:** Tomasz Raczyński, Daniel Janczak, Jerzy Szałapak, Piotr Walter, Małgorzata Jakubowska

**Affiliations:** 1 Institute of Metrology and Biomedical Engineering, Faculty of Mechatronics, Warsaw University of Technology, 00-661 Warsaw, Poland; daniel.janczak@pw.edu.pl (D.J.); jerzy.szalapak@pw.edu.pl (J.S.); piotr.walter.dokt@pw.edu.pl (P.W.); malgorzata.jakubowska@pw.edu.pl (M.J.); 2 Central Laboratory, Centre for Advanced Materials and Technologies (CEZAMAT), 02-822 Warsaw, Poland

**Keywords:** wearable electronics, printed electronics, thermal transfer, carbon elastic heaters, screen-printing

## Abstract

Wearable electronics are new structures with a wide range of possible applications. This study aims to analyze the effects of hot pressing in thermal transfer of different carbon-based composites as a new application method of screen-printed electronics on textiles. Flexible heaters were screen-printed on polyethylene terephthalate PET foil with composites based on graphene, carbon black, and graphite with different wt.%, measured and then hot pressed to measure and analyze differences. Research showed that the hot pressing process in thermal transfer resulted in decreased electrical resistance, increased power, and higher maximal temperatures. Best results were achieved with composites based on 12 wt.% graphene with sheet resistance lowered by about 40% and increased power by about 110%. This study shows promise for thermal transfer and screen-printing combination as an alternative for creating flexible electronics on textiles.

## 1. Introduction

Driven by marketing, trends among the youth, and high capital, wearable electronics are becoming one of the most popular and highly discussed topics [1]. Many companies are already in this market, offering smartwatches, pulsometers, smart bands, and other smart accessories. Offered products already evolved beyond medical purposes, entering a sphere of commercial, entertainment, or industrial applications [2].

With an increase in demand, research and development of those technologies became more focused. The development of digital medicine was one of the most important driving factors behind this change. Due to a need to monitor patients over prolonged periods and sometimes even do it remotely [3], wearable electronics moved from carried elements to integrated electronics. However, this transition comes with its difficulties. Those new devices must be flexible, convenient to use, miniaturized, and even aesthetically appealing.

The niche thus created has been filled by thick-film electronics. Screen printing is an example of the implementation of this technology. It allows flexibility in designing patterns and using different materials while maintaining mechanical strength without sacrificing electrical performance, dimensions, and convenience [4]. However, direct printing on fabric comes with its own set of complications. The un-uniformity and pliability of substrate hinders the printing process at the cost of repeatability and the ability to print small-sized structures. Indirect printing, in which the printing process is separated from transferring the print to the fabric, might solve this problem, allowing for increased accuracy and repeatability. However, this is a little researched method that is still in the development phase.

In the present paper, the authors focused on flexible heaters as a potential application for flexible electronics. Currently, there is an increasing demand for flexible heaters. They are used in the semiconductor industry [5] as portable heaters for personal use [6] or for reheating food [7]. They work as resistance heaters, which allows for precise temperature control by changing the voltage. Classically used heaters are made of rare materials or lack the flexibility needed for more demanding applications [8].

For this reason, composite solutions containing nanomaterials are being explored. It allows the use of flexible substrates and vehicles while maintaining low resistance. The primary materials chosen are silver nanowires or carbon-based nanomaterials. Currently researched materials and methods are presented in Table 1. The use of silver nanomaterials results in low resistances, but carbon-based materials permit the production of biodegradable components and lower production costs. The method of laser pattering resulted in high temperatures, but such a process is slow and costly, not allowing for mass production. Of all those methods, screen printing allows for the most effortless mass production of such heaters. The main parameter of electric heaters is resistance. Change of this parameter during the design process allows for direct control of the power of a heater. Based on the available literature, heaters resistance should be under 1000 Ω/sq.

However, to manufacture such wearable electronics in industrial quantities, it is necessary to simplify the process and ensure repeatability. This can be achieved by using a combination of screen printing and thermal transfer. Directly screen-printing electrical structures on textiles comes with many different complications. The elasticity of textiles as a substrate makes it difficult to position for multi-layered structures, and high permeability makes it impossible to mount using a vacuum table commonly used in the screen-printing process. Thermal transfer separates the process of printing the electronics from applying them to the fabric, allowing the use of less complicated equipment and the exploitation of existing processes. The use of thermal transfer in creating flexible electronics on fabrics is a very new field. Currently, printing conductive surfaces using ink containing silver nanowires [9], a body heat absorber integrated into clothing [10], an antenna for signal transmission made of carbon inks [11], and a piano integrated into fabric [12] have been investigated. Due to the novelty of this technology and its potential for mass production, it requires further investigation.

This work investigates different carbon-based composites for flexible heaters and the effects of hot pressing in the thermal transfer on their thermal and electrical properties. The results of this study will help to understand the process of thermal transfer better and help to facilitate further research in textile integrated electronics.

## 2. Materials and Methods

### 2.1. Materials

Three types of material were chosen for conductive components of produced composites. Graphene nanoplatelets type C (GNP C), acquired commercially from CheapTubes, Grafton, MA, USA, was chosen based on previous research [19,20,21]. Following successful results in conductive composites research [22,23,24,25], conductive soot, purchased as Carbon Black (CB) from Graphene Laboratories Inc., Calverton, NY, USA, was chosen. The last tested material was graphite, proven for its conductivity [26,27,28] and purchased as MG 1596 from Termoplastik, Bydgoszcz, Poland. Different physical properties of those materials: dimensions and volume, are presented in Table 2.

To produce the organic base for flexible heaters, thermoplastic polyurethane polymer (TPU) was chosen. It was characterized by 37 Shore hardness, the density of 1.18 g/cm^3^, shear resistance of 27 N/mm, the tensile strength of 12 MPa, and elongation before breaking of 1150%. It was commercially acquired from the BASF company. It was used as a solution in a mixture of Dimethylformamide (DMF).

All structures were printed on PET foil with a thickness of 125 µm.

### 2.2. Preparations

To produce the vehicle for carbon-based composite, a solution containing 20 wt.% of TPU in DMF was prepared to ensure rheological properties appropriate for screen printing [4]. The dissolution process was conducted at a temperature of 30 °C using a magnetic mixer for two hours.

Carbon materials in desired amounts were mixed with a vehicle in a speed mixer until achieving a homogenous composite. Composites were prepared using GNP C (10–20 wt.%), CB (10–17 wt.%), and Graphite (20–40 wt.%). Amounts of the carbon material were chosen to achieve the highest density of carbon materials with rheological properties still suitable for screen printing.

**Figure 1:**
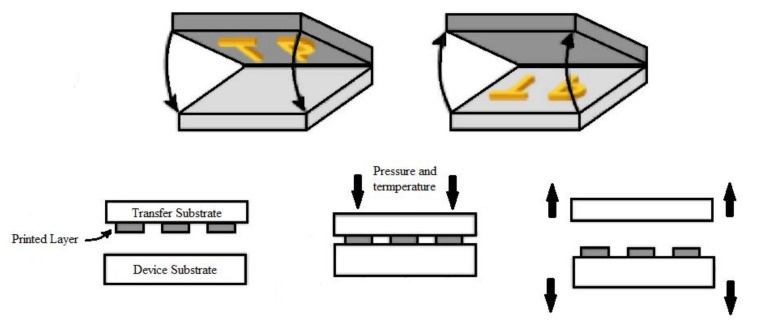
Schematic of the thermal transfer process.

Composites were screen printed using 68T polyester mesh creating 10 × 28 mm heaters, dried for 20 min at 120 °C. Samples were then measured, hot pressed, and then measured again. They were pressed using a heat press with a temperature of 180 °C for 60 s with a force of 150 g/cm^2^. The schematic of this process is shown in Figure 1.

## 3. Results and Discussion

**Figure 2:**
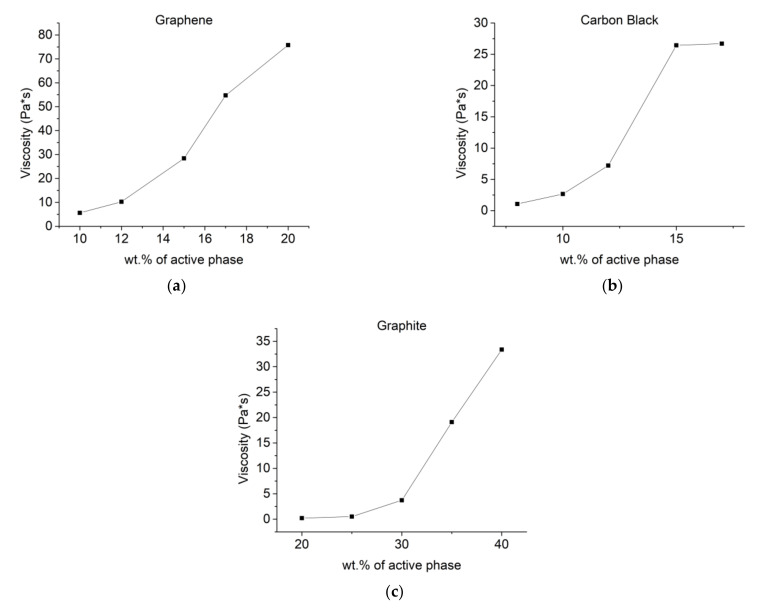
Rheological measurements of prepared composites displaying the relationship between wt.% of active phase and viscosity with the shear rate of 50 1/s for composites based on: (**a**) Graphene; (**b**) Carbon black; (**c**) Graphite.

To check the viability of prepared composites for screen printing, their rheological properties were measured. They were tested using the cone-plate method with an increasing shear rate. Results of those measurements are displayed in Figure 2. The viscosity of prepared pastes was increasing with higher wt.% of active phase as expected. Due to the high viscosity of composites containing 18 wt.% and 20 wt.% graphene those were not possible to screen-print.

**Figure 3:**
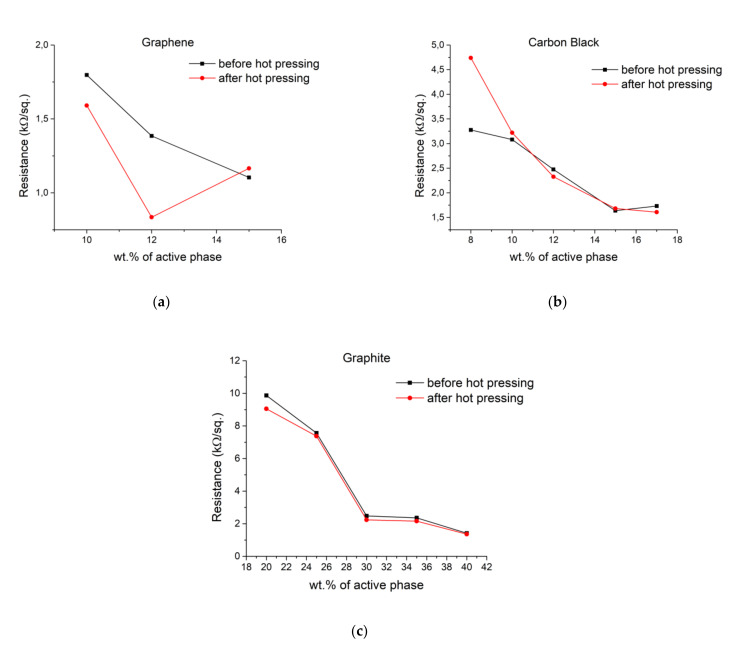
Comparison of resistances before and after a hot pressing process for heaters screen-printed with different compositions of pastes based on: (**a**) Graphene; (**b**) Carbon black; (**c**) Graphite.

After successful screen-printing, the resistance of all heaters (n = 20) was measured. Next, they were hot-pressed and measured again. Results of those measurements are presented in Table 3, with additional comparisons of results before and after the hot pressing process in Figure 3.

While heaters made using graphite and carbon show no changes, those made with graphene display lower resistances after hot pressing, decreasing their value by about 40%. The high surface area of graphene should allow for the additional compression of composite, increasing numbers of connections between single platelets and thus resulting in lower electrical resistance. Janczak et al. [12] observed such phenomena, but with no change of resistance for carbon black, which also has a high surface area, such change in resistance comes from the shape of individual particles of conductive phase. While carbon black and graphite could be categorized as amorphic, graphene is in the shapes of platelets. During high pressure and temperature, TPU becomes malleable, allowing for a change of orientation of graphene nanoplatelets, positioning them in sheets, and lowering their resistance. Those changes are not visible in composites with 15 wt.% of graphene. This is due to the maximum filling threshold of graphene in composite, not allowing for compression on change in orientation. Using TPU and a combination of increased pressure and temperatures, those changes in the orientation of graphene will be persistent between thermal expansion and compression cycles of heaters.

**Figure 4:**
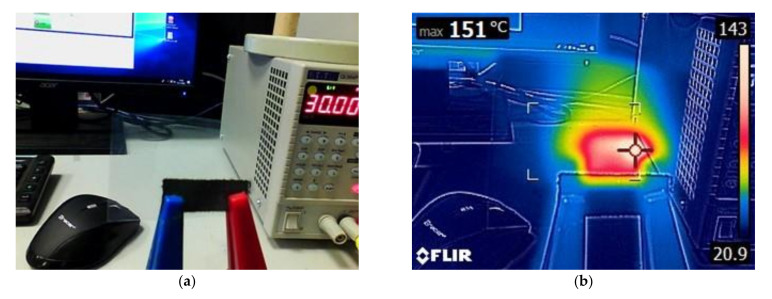
Photos showing measurement of heater screen-printed with 12 wt.% graphene paste taken with a thermal camera: (**a**) regular picture, (**b**) thermal picture.

To test the proof of concept, a heater based on 12 wt.% graphene paste was connected to the power supply and measured with a Flir E5 thermal camera with a measuring range of 0–200 °C (Figure 4).

**Figure 5:**
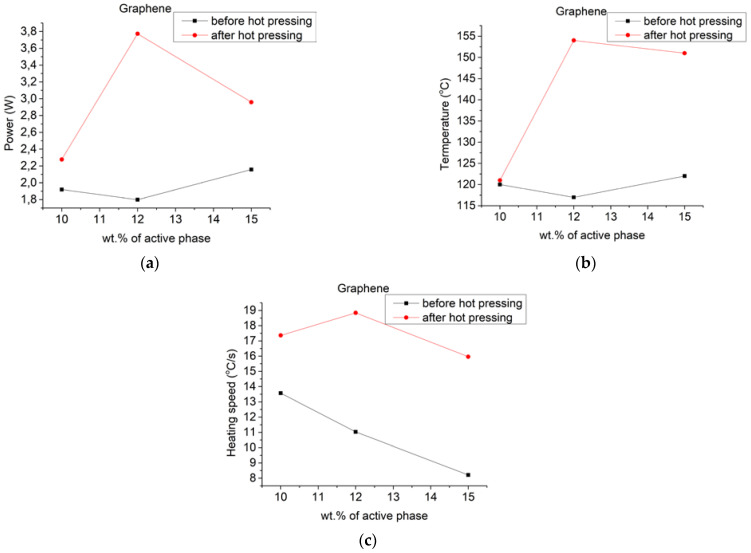

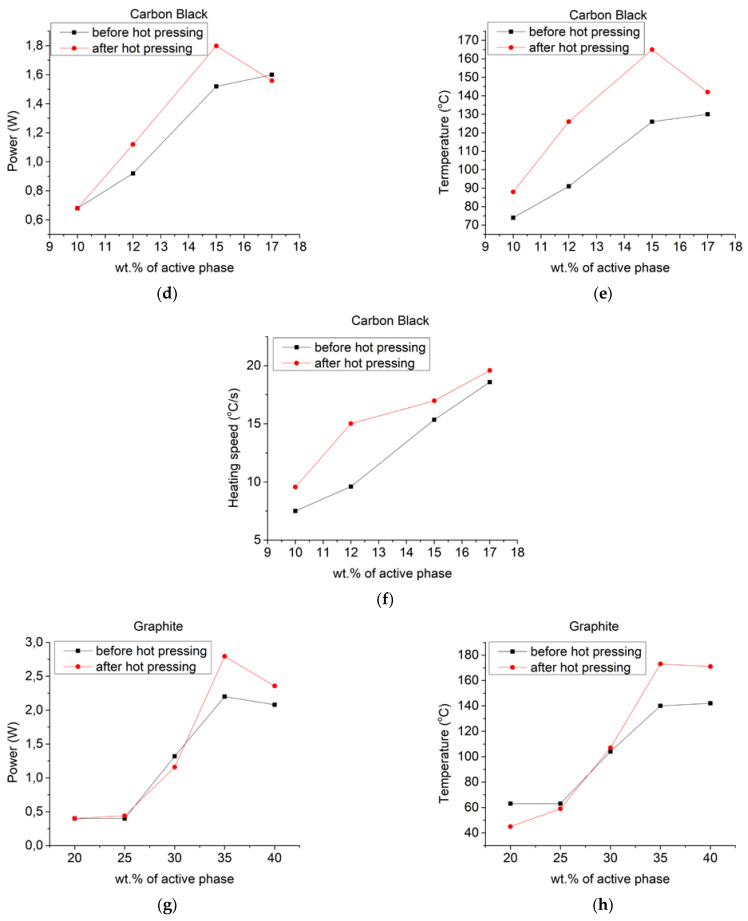

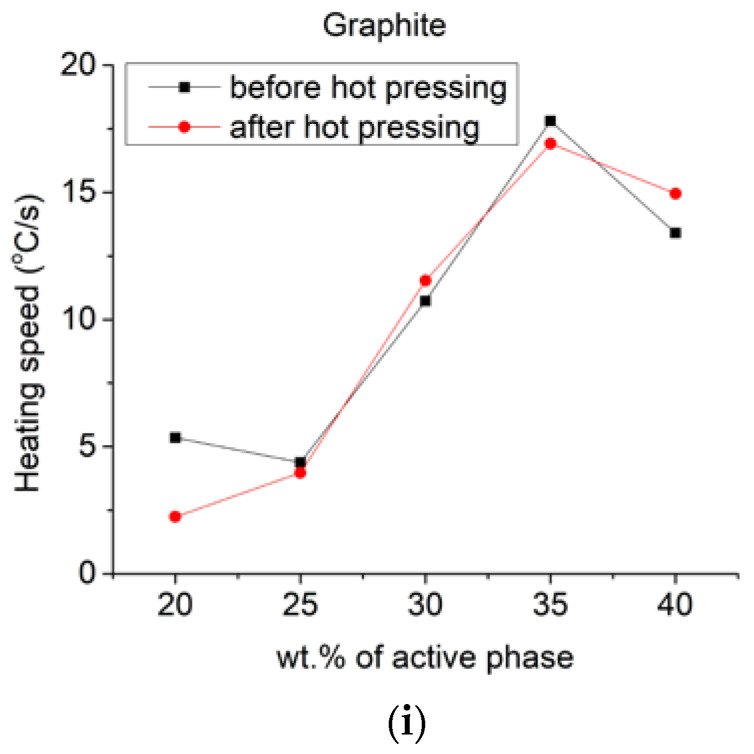
Results of evaluation of thermal properties of heaters made (**a**–**c**) with graphene nanoplatelets, (**d**–**f**) carbon black and (**g**–**i**) graphite showing the relation between wt.% of functional phase and: (**a**,**d**,**g**) power; (**b**,**e**,**h**) resulting temperatures; (**c**,**f**,**i**) heating speed; before and after hot pressing with an applied voltage of 40 V.

To evaluate the thermal properties of created heaters, they were connected to a power supply with a voltage value of 40 V. During this measurement, resistance, power, and temperature were collected in relation to time. It allowed to discern multiple characteristics: heaters power, highest achieved temperature and heating speed. Results of those findings are displayed in Figure 5.

From those results, the most noticeable changes were for heaters made with graphene. The best results were obtained for composites containing 12 wt.% graphene with an increase in power of around 110%. Noteworthy was an increase in highest temperatures for 12 wt.% and 15 wt.% with differences up to 60 °C and a constant increase in heating speed for all values resulting in more efficient and more dynamic heaters. As previously stated, such changes are an effect of the very high surface area of graphene nanoplatelets, that after compression increase in density, an increasing number of electrical connections between single platelets. Carbon black-based heaters showed a slight increase in power and heating speed with a more significant change of the highest temperature of about 40 °C with almost no changes in electrical resistance. Heaters containing graphite showed a slight increase in power and highest temperatures for 35 wt.% and 40 wt.% with no noticeable heating speed changes. For carbon black and graphite, an increase in temperature can be attributed to increased density of composite after hot pressing, and lack of change in electrical resistance is mainly due to being unable to reach lower resistance values for this type of materials.

## 4. Conclusions

TPU based composites using graphene nanoplatelets, carbon black, and graphite were prepared. The rheological properties of those composites were measured to ascertain suitability for screen-printing. Heating structures were screen-printed using chosen pastes. A series of tests were made before and after the hot-pressing process to measure changes in resistance, power, temperature, and heating speed.

Of all tested materials, the best results were achieved using composites containing 12 wt.% graphene nanoplatelets. There was a decrease of sheet resistance of around 40% and an increase in power from 1.798 W to 3.773 W. An increase in heating speed was also observed.

Performed tests showed that heat pressing in the thermal transfer process could improve screen-printed heaters’ electrical and thermal properties. Conducted research shows that screen-printing and thermal transfer, due to its low production costs and results comparable to existing research, can have a high potential for applications in textronics. In addition, there is a possibility that the process of heat pressing can improve the electrical properties of other screen-printed composites.

## Figures and Tables

**Table 1:** Comparison of Materials, Production Methods, Electrical and Thermal Properties of Existing Research into Flexible Heaters.

Material	Production Method	Resistance, Ω/sq.	Voltage, V	Temperature, °C	Source
Graphene Oxide	Laser patterning	1000	18	247.3	[13]
Graphene Oxide, silver particles	Drop coating	158	15	160	[14]
Single-walled carbon nanotubes	Doctor-blade	93	7	140	[15]
Silver nanowire	Polymer casting	30	10	200	[6]
Silver nanowire	Polymer casting	30	16	131	[16]
Silver nanowire	Screen-printing	380	40	99	[8]
Silver fractal dendrites	Screen-printing	0.83	2	107.4	[17]
Graphene, carbon nanotubes	Screen-printing and lamination	500	12	57	[18]

**Table 2:** Physical Properties of Chosen Carbon Materials.

Material	Diameter, µm	Thickness, nm	Surface Area, m^2^/g
GNP C	2	8–15	500–700
CB	0.3	30	254
Graphite	10	10,000	~1.5

**Table 3:** Detailed Results of Resistance Measurements Before and After a Hot Pressing Process for Heaters Screen-Printed with Different Composites.

Sample Name	Resistance Before Hot Pressing, kΩ/sq.	Resistance After Hot Pressing, kΩ/sq.
GNPC_10	1.80 ± 0.4085	1.59 ± 0.13608
GNPC_12	1.38 ± 0.48518	0.84 ± 0.31876
GNPC_15	1.10 ± 0.31062	1.17 ± 0.31077
CB_10	3.08 ± 0.12073	3.22 ± 0.38048
CB_12	2.48 ± 0.23937	2.33 ± 0.2547
CB_15	1.64 ± 0.23031	1.68 ± 0.22878
CB_17	1.73 ± 0.23592	1.61 ± 0.25223
G_20	9.87 ± 1.33997	9.05 ± 1.87091
G_25	7.56 ± 1.45489	7.37 ± 1.61598
G_30	2.48 ± 0.48055	2.24 ± 0.23633
G_35	2.36 ± 1.37034	2.16 ± 1.21135
G_40	1.42 ± 0.43242	1.36 ± 0.19569

## Data Availability

All the data is available within the manuscript.

## References

[1] Matthew R., Dutta J., Maheswar R., Ahmed K. (2021). Intelligent Wearable Electronics: A New Paradigm in Smart Electronics. Challenges and Solutions for Sustainable Smart City Development EAI/Springer Innovations in Communication and Computing.

[2] Hayward J. Wearable Technology Forecasts 2019–2029; Report for IDTechEx; IDTechEx: 2019. https://www.idtechex.com/en/research-report/wearable-technology-forecasts-2019-2029/680.

[3] Elenko E., Underwood L., Zohar D. (2015). Defining Digital Medicine. Nat. Biotechnol..

[4] Janczak D., Zych M., Raczyński T., Dybowska-Sarapuk Ł., Pepłowski A., Krzemiński J., Sosna-Glłȩbska A., Znajdek K., Sibiński M., Jakubowska M. (2019). Stretchable and Washable Electroluminescent Display Screen-printed on Textile. Nanomaterials.

[5] Cho N.-I., Kim M.C. (2005). Preparation of Cr-Si Multilayer Structures for Thin Film Heater Applications. Thin Solid Films.

[6] Hong S., Lee H., Lee J., Kwon J., Han S., Suh Y.D., Cho H., Shin J., Yeo J., Ko S.H. (2015). Highly Stretchable and Transparent Metal Nanowire Heater for Wearable Electronics Applications. Adv. Mater..

[7] Shin K.Y., Hong J.Y., Lee S., Jang J. (2012). High Electrothermal Performance of Expanded Graphite Nanoplatelet-based Patch Heater. J. Mater. Chem..

[8] He X., He R., Lan Q., Wu W., Duan F., Xiao J., Zhang M., Zeng Q., Wu J., Liu J. (2017). Screen-Printed Fabrication of PEDOT:PSS/Silver Nanowire Composite Films for Transparent Heaters. Materials.

[9] Maheshwari N., Abd-Ellah M., Goldthorpe I.A. (2019). Transfer Printing of Silver Nanowire Conductive Ink for e-textile Applications. Flex. Print. Electron..

[10] Elmoughni H.M., Menon A.K., Wolfe R.M.W., Yee S.K. (2019). A Textile-Integrated Polymer Thermoelectric Generator for Body Heat Harvesting. Adv. Mater. Technol..

[11] Pulanthran K., Jizat N.M., Islam M.S. A Low-cost Textile Antenna using Thermal-transfer Printing. Proceedings of the 16th IEEE International Colloquium on Signal Processing and its Applications, CSPA 2020.

[12] Kost L., Pavec M., Michal D., Moravcova D., Soukup R., Hamacek A. E-textile Piano Fabricated using Several Textile Technologies. Proceedings of the International Spring Seminar on Electronics Technology.

[13] Zhang T.Y., Zhao H.M., Wang D.Y., Wang Q., Pang Y., Deng N.Q., Cao H.W., Yang Y., Ren T.L. (2017). A super flexible and custom-shaped graphene heater. Nanoscale.

[14] Lin S.Y., Zhang T.Y., Lu Q., Wang D.Y., Yang Y., Wu X.M., Ren T.L. (2017). High-Performance Graphene-Based Flexible Heater for Wearable Applications. RSC Adv..

[15] Kim Y., Lee H.R., Saito T., Nishi Y. (2017). Ultra-Thin and High-Response Transparent and Flexible Heater Based on Carbon Nanotube Film. Appl. Phys. Lett..

[16] Pyo K.-H., Kim J.-W. (2016). Transparent and Mechanically Robust Flexible Heater Based on Compositing of Ag Nanowires and Conductive Polymer. Compos. Sci. Technol..

[17] Zeng P., Tian B., Tian Q., Yao W., Li M., Wang H., Feng Y., Liu L., Wu W. (2019). Screen-Printed, Low-Cost, and Patterned Flexible Heater Based on Ag Fractal Dendrites for Human Wearable Application. Adv. Mater. Technol..

[18] Park H.K., Kim S.M., Lee J.S., Park J.H., Hong Y.K., Hong C.H., Kim K.K. (2015). Flexible Plane Heater: Graphite and Carbon Nanotube Hybrid Nanocomposite. Synth. Met..

[19] Młyńczak M., Zyliński M., Janczak D., Jakubowska M., Niewiadomski W., Cybulski G. Graphene electrodes for long-term impedance pneumography—A feasibility study. Proceedings of the European Medical and Biological Engineering Conference.

[20] Dybowska-Sarapuk L., Janczak D., Podsiadly B., Jakubowska M., Sloma M. (2019). Electrical and Rheological Percolation Threshold of Graphene Pastes for Screen-Printing. Circuit World.

[21] Pepłowski A., Rathi S., Piotrkowski B., Ziółkowski R., Janczak D., Krzemiński J., Brosch M., Jakubowska M. (2020). Electrochemistry of Graphene Nanoplatelets Printed Electrodes for Cortical Direct Current Stimulation. Front. Neurosci..

[22] Bourrat X. (1993). Electrically Conductive Grades of Carbon Black: Structure and Properties. Carbon.

[23] Gubbels F., Jerome R., Teyssie P., Vanlathem E., Deltour R., Calderone A., Parents V., Bredas J.L. (1994). Selective Localization of Carbon Black in Immiscible Polymer Blends: A Useful Tool To Design Electrical Conductive Composites. Macromolecules.

[24] Duan L., Spoerk M., Wieme T., Cornillie P., Xia H., Zhang J., Cardon L., D’hooge D.R. (2019). Designing Formulation Variables of Extrusion-Based Manufacturing of Carbon Black Conductive Polymer Composites for Piezoresistive Sensing. Compos. Sci. Technol..

[25] Phillips C., Al-Ahmadi A., Potts S.J., Claypole T., Deganello D. (2017). The Effect of Graphite and Carbon Black Ratios on Conductive Ink Performance. J. Mater. Sci..

[26] Sengupta R., Bhattacharya M., Bandyopadhyay S., Bhowmick A.K. (2011). A Review on the Mechanical and Electrical Properties of Graphite and Modified Graphite Reinforced Polymer Composites. Prog. Polym. Sci..

[27] Kalaitzidou K., Fukushima H., Drzal L.T. (2007). Multifunctional Polypropylene Composites Produced by Incorporation of Exfoliated Graphite Nanoplatelets. Carbon.

[28] Zheng W., Wong S.C. (2003). Electrical Conductivity and Dielectric Properties of PMMA/expanded Graphite Composites. Compos. Sci. Technol..

